# miR-664b-3p inhibits colon cell carcinoma via negatively regulating Budding uninhibited by benzimidazole 3

**DOI:** 10.1080/21655979.2022.2036400

**Published:** 2022-02-13

**Authors:** Liang-Yu Zhao, Guo-Jun Xin, Yuan-Yuan Tang, Xiao-Fei Li, Yu-Zhen Li, Ning Tang, Yu-Hong Ma

**Affiliations:** a Department of Gastrointestinal Surgery, People’s Hospital of Ningxia Hui Autonomous Region, the First Affiliated Hospital of Northwest Minzu University, Yinchuan, China; b Department of Hepatobiliary Surgery, People’s Hospital of Ningxia Hui Autonomous Region, the First Affiliated Hospital of Northwest Minzu University, Yinchuan, China; c Department of Gastroenterology, People’s Hospital of Ningxia Hui Autonomous Region, the First Affiliated Hospital of Northwest Minzu University, Yinchuan, China; d Department of Digestive Endoscopy Center, People’s Hospital of Ningxia Hui Autonomous Region, the First Affiliated Hospital of Northwest Minzu University, Yinchuan, China

**Keywords:** miR-664b-3p, colon cancer, BUB3 protein

## Abstract

MiR-664b-3p has been reported to play a crucial role in cancer progression. This research explores the biological effect and molecular mechanisms of miR-664b-3p in cell proliferation, apoptosis, migration, and invasion of colon cancer. The expression level of miR-664b-3p and Budding uninhibited by benzimidazole 3 (Bub3) in colon cancer cell lines and tissues were detected and analyzed using quantitative real-time PCR and bioinformatics method. The Western blot measured the expression level of proliferation-related, migration-related, and apoptosis-related proteins. CCK-8 assessed cell viability, and the cell proliferation, migration, and invasion were detected by the Edu assay, wound-healing assay, and transwell assay, respectively. Annexin/propidium iodide (PI) assays detected apoptosis of cells. The target of miR-664b-3p was predicted by bioinformatics methods and then validated by gene engineering technology. MiR-664b-3p was downregulated in colon cancer tissues and cells. The cell proliferation, migration, and invasion of cells were inhibited after transfecting by miR-664b-3p mimics, whereas apoptosis was promoted. Over-expression of miR-664b-3p could reduce the expression of proliferation-promoted proliferating cell nuclear antigen (PCNA), proliferation marker protein Ki-67 (Ki-67), migration-promoted Cyclooxygenase-2 (COX-2), Matrix Metallopeptidase 2 (MMP-2), and Matrix Metallopeptidase 9 (MMP-9), and apoptosis-inhibited protein (Bcl-2) while increasing the expression of apoptosis-promoted BCL2-Associated X Protein (Bax), caspase-3, and caspase-9 proteins. The study indicated that miR-664b-3p plays a significant role in colon cancer and could regulate the progression of colon cancer tumor growth by suppressing the expression of BUB3 protein. These findings provide a novel strategy to screen and treat colon cancer.

## Graphical abstract

**Figure:**
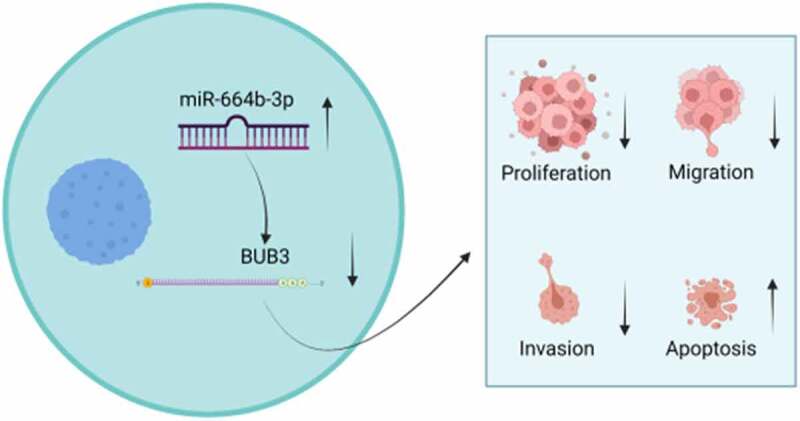

## 1. Introduction

Colon cancer (CC) has become one of the most malignant diseases that seriously threaten human life in many countries, especially in developing countries [1–3]. Several pieces of evidence proved that timely diagnosis has a highly significant impact on the prognosis of the disease [4,5]. Although the existing screening and treatment method in the clinic could reduce the incidence of CC, the incidence rate keeps increasing. It has been predicted that CC patients will rise by 70% worldwide by 2030 [6,7]. Thus, a novel biomarker is necessary for diagnosing appropriately, increasing the possibility of recovery from the disease.

More recently, the microRNAs (miRNAs), evolutionarily conservative non-coding RNA molecules with about 19–25 nucleotides in length, has been demonstrated that they be blessed with the ability to regulate the related-gene expression by binding to the 3’-untranslated regions (3’ UTR) on mRNAs [8,9], participating in the progression and development of the tumor. It has been reported that these miRNAs control approximately 35% of the expression of human genes and proteins and thus play a critical role in regulating the proliferation [10], differentiation [11], and apoptosis of cells [12]. Wang *et al*. found that miRNA-129-5p exosomes induced apoptosis in colon cancer cells while inhibiting their proliferation, migration, and invasion [13]. It has been reported that up-regulation of miR-664b-3p could inhibit the expression of glycogen synthase kinase-3β interaction protein (GSKIP) and then suppress the Wnt/β-catenin signaling pathway, repressing the proliferation and invasion of hepatocellular carcinoma [14]. Moreover, low expression of miR-644b-3p was closely related to the metastatic risk in pancreatic neuroendocrine tumors [15]. However, the relationship between miR-644b-3p and the occurrence of CC and its mechanism is still unknown.

BUB (Budding uninhibited by benzimidazole) protein family is part of a large multiprotein kinetochore complex, regarded as a vital component of the checkpoint regulation pathway [16]. BUB3, a protein encoded by the A.thaliana genome, is an evolutionary conservation protein in animals and fungi and constitutes the spindle assembly checkpoint (SAC), which ensures that all sister chromatids are attached to kinetochore fibers originating from opposite poles of the bipolar spindle before anaphase onset [17,18]. Previous studies have identified that overexpression of the BUB3 could increase the proliferation of oral squamous cell carcinoma cells. Meanwhile, it has also been observed that mutations in BUB3 could cause variegated mosaic aneuploidy and increase the risk of colorectal cancer [19,20]. Nevertheless, the significance of BUB3 in CC has not been completely clarified.

In this study, We hypothesized that miR-664b-3p could regulate the progression of colon cancer tumor growth by inhibiting the expression of Bub3 protein. To test our hypothesis. We studied the expression of miR-664b-3p in CC patients and CC cells and the effects of miR-664b-3p expression level on CC cell proliferation, invasion, migration and apoptosis and its regulatory mechanism. Our study provides a new strategy for screening and treating colon cancer.

## 2. Materials and methods

### 2.1 Bioinformatics analysis

TCGA- colon adenocarcinoma (COAD) (https://portal.gdc.cancer. gov/) database was used to obtain the sequence of miRNA and RNA and subjected to differential analysis using the ‘edgeR’ package. ENCORI and miRWalk were used to predict the miRNA which regulates the BUB3 protein. The intersection of these two databases was selected. The FunRich function was used to sort proteins closely related to the cell cycle and mitosis.

### 2.2 Cell Culture

NCM460, DLD-1 and HCT116 lines were obtained from Green Flag (Shanghai) Biotechnology Development Co., LTD. NCM460 was incubated with Eagle’s Minimum Essential Medium (EMEM; Catalog No. 30–2003) containing 10% Fetal Bovine Serum (FBS). Then, the cell plate was placed in the cell incubator. DLD-1 and HCT116 cell lines were incubated in McCoy’s 5a Medium Modified (Sigma No. 256124) that contained 10% FBS, while SW620, SW115 and SW480 cell lines were grown in Leibovitz’s L-15 Medium (Sigma No. 26621) that contained 10% FBS, and all cells were cultured in a humid atmosphere with 5% CO_2_ at 37°C. The cell reaching the logarithmic phase was harvested in the subsequent experiment [21].

### 2.3 Cell transfection

Harvested cells were plated onto the 6-well plate at a concentration of 5 × 10^5^ cells per well and cultured in an incubator. Before transfection, Opti-MEM was used to dilute the NC-mimics, NC-inhibitor, miR-664b-3p mimics, miR-664b-3p-inhibitor, pc-NC, pc-BUB3, and Lipo200 to 20 nM. Then, the Lipo 2000 and these mimics or inhibitors were added to the plate for 4 h at 37°C. Afterward, cells were cultured in a humid atmosphere under a 5% CO_2_ at 37°C after being washed twice with PBS and were collected after 48 h [21].

### 2.4 Real-time quantitative PCR (qRT-PCR)

According to the protocol, the Trizol reagent (Invitrogen) extracted the total RNA of CC cells. Next, the complementary DNA (cDNA) was obtained by transcribing RNA. RNA and cDNA quality was determined by Bioanalyzer (Agilent). The commercialized cDNA Reverse Transcription kit (Applied Biosystems, USA) was used. The level of qRT-PCR was assessed with the MicroRNA Assay and TaqMan® Universal PCR Master Mix (Applied Biosystems, USA) under the thermocycler conditions: 95°C for 5 min, and 40 cycles of 95°C for 5 s and 60°C for 31s, followed by 75°C for 40s. Besides, qRT-PCR was implemented to measure the level of mRNA and miRNA under the specific thermocycler conditions according to the agreement of the commercialization kit [21]. The primers were obtained from TAKARA (Beijing, China) and listed in Table 1.

### 2.5 Western blot

Proteins of cells after transfection were obtained according to the reported method [22]. The protein concentration was assessed by Bicinchoninic Acid Assay (BCA) Protein Assay kit (BD Biosciences) and denatured with SDS-PAGE loading buffer for 5 min in boiling water. Protein samples were detached on SDS-PAGE (10%) (Sigma, USA) and transferred onto the membranes that are polyvinylidene difluoride (PVDF) (Millipore, USA). The membranes were blocked for 2 h at room temperature using 5% nonfat milk (BD, New Jersey, USA). Then the primary antibodies were added to incubate with the membrane at the 4°C overnight Primary antibodies used for analysis were: PCNA (Abcam 218311), Ki-67 (Abcam 134175), COX-2 (Abcam 96723), MMP-2 (Abcam 32419), MMP-9 (Abcam 92486), Bax (Abcam 32503), Bcl-2 (Abcam 185002), caspase-3 (Abcam 13847), BUB3 (Abcam 181602), and caspase-9 (Abcam 219590). After that, the Tris Buffered Saline Tween (TBST) was used to wash the incubated membranes five times. Then, the Horseradish peroxidase (HRP)-conjugated goat anti-rabbit/mouse IgG (Cell Signaling technologu, MA) was added to the mixture at 20°C for 2 h. Finally, the membranes were washed 10 min five times in TBST and investigated with a substrate super ECL Plus solution (Beyotime, Shanghai, China). The image was captured with Image J (BIO-RAD, USA).

### 2.6 Cell Counting Kit-8 (CCK-8) assay

HCT-116 and DLD-1 cells were harvested using the digestion and were plated into the 96-well plates (5 × 10^5^ cells/mL each well) and incubated for 0, 24, 48 and 72 h. Next, the 10 uL of CCK-8 solution (Beyotime, Shanghai, China) was added into each well, and the mixture was placed in the incubator at 37°C for 4 h. At last, the micro-oscillator (Shanghai Yairong Biochemical Instrument Factory, Shanghai, China) was used to shake the mixture for 10 min. The microplate reader (Thermo, Waltham, USA) was used to measure the absorbance at 450 nm [23].

**Table 1.** Primer sequences used in the study

Name	Forward primer (5‘-3’)	Reverse primer (5‘-3’)
miR-664b-3p	TGGGCTAAGGGAGATGATTG	TGTAGGCTGGGAGGCAAA
BUB3:	TGATTAGGTGGACTTGGGTTTT	AATGATGGGACTACGCTTGC
CENPL	GGCGAGCAAAGCCAAAAT	GCCGAGCCACTTAACCACTAT
CDK2	ACCTAATAGGCTGGGAGACTGA	GGACAAACAAGGGGACTGG
CDC27	GACAGTTCTTACGGAAACACCC	AAGAGCTGCTGGTCCTCCTA
DSN1	CACTACATTCAACGAACAAGGG	CAAAAGACGCATTTCCAACC
DYRK1A	GCCTCTTTCCAGTTCTCCAC	GCCCCTAACCCTGCTTCTA
U6	AACGCTTCACGAATTTGCGT	AACGCTTCACGAATTTGCGT
GAPDH	ACCCAGAAGACTGTGGATGG	CACATTGGGGGTAGGAACAC

### 2.7 Dual-luciferase reporter gene assay

The wild-type (WT) BUB3 3ʹUTR, which could connect with miR-644b-3p and the mutant (MUT)BUB3 3ʹUTR were formed by site-directed mutagenesis method. The cells, including HCT-116 and DLD-1, were transfected by the BUB3 WT/BUB3-MUT vectors together with the NC mimics or miR-664b-3p mimics, respectively. Then, the Reporter Assay Kit (Thermo Fisher FD1703) was used to detect the fluorescence intensity, which indicated the luciferase activities. Briefly, the cell was cracked using the 1× Passive lysis buffer after transfection and incubation for 48 h. The experiment was performed in triplicate [21].

### 2.8 Detection of cell apoptosis

The Annexin V/PI protocol was used to detect the apoptosis cell in this study. Briefly, cultured cells were harvested and centrifuged at 1000 rpm for 5 min. After washing twice by cold PBS, the IX solution was used to mark and resuspend collected cells. Then, the 5 ul of diluted AnnexinV- Fluorescein Isothiocyanate (FITC) and PI were added to the labeled cell. The mixture was incubated at room temperature for 15 min without light. The flow cytometry detected the fluorescence intensity (FACSCalibur, BD Biosciences, San Jose, CA, USA) [24].

### 2.9 Wound-healing migration assay

Cultured cells were harvested and seeded into the 24-well plate, and the content of each well was 1 × 10^5^. After that, the tweezers were used to produce the scratch, which possessed a 500 μm of wide. The plated was incubated at 37°C for additional time. The microscope was used to observe the migration rate of cells after 24 h [23].

### 2.10 Cell transwell assays

Transwell chambers were used to evaluate the invasion potential of cells in the study. Briefly, cells (2 × 10^5^) were plated to the upper chamber coated with Matrigel (BD, Biosciences) and cultured in serum-free media, while 10% FBS dissolved in the media was added to the bottom chamber to induce the migration of upper cells. The symptom was incubated for 48 h. Afterward, the cotton swab removed the noninvading cells from the upper chambers. The 95% ethanol was used to fix the cells that invaded to the bottom, staining with 0.1% crystal violet, and then counted at five randomly selected fields using a microscope (Olympus Optical Co., Ltd., Tokyo, Japan) [24].

### 2.11 Statistical analysis

The results were performed as mean ± SEM. The Kolmogorov-Smirnov test was used to analyze the normal distribution of data. The experiment in each group was repeated at least 3 times. Statistical analysis was exerted using GraphPad Prism 8.0 software. Unpaired Student’s t-tests (only two groups) and One-Way Analysis of variance (ANOVA) test (more than two groups) assessed P values. A probability value (P) less than 0.05 was considered statistically significant.

## 3. Results

In this study, we hypothesized that miR-664b-3p regulates the progression of colon cancer by inhibiting the expression of Bub3 protein. We studied the expression of miR-664b-3p in CC patients and CC cells. In addition, we also studied the effects of miR-664b-3p on the proliferation, invasion, migration and apoptosis of CC cells and their regulatory mechanism.

### 3.1 miR-664b-3p was down-regulated in the colon adenocarcinoma (COAD) tissues and colon cancer (CC) cells

**Figure 1.**
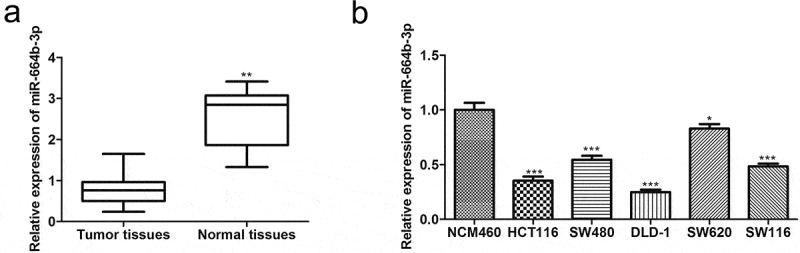
(a) The expression level of miR-664b-3p in COAD tissues, the analysis was obtained from Starbase. **P < 0.01 vs. Tumor tissues. (b) The expression level of miR-644b-3p in different colon cancer cell lines. *P < 0.05, ***P < 0.001 vs. NCM460 cell lines.

In order to investigate the differential expression status of miRNAs between normal and COAD tissues, normal samples (n = 8) and COAD tissues (n = 450) were obtained and analyzed by the ENCORI database that is updated faster than other kinds of databases. As shown in Figure 1(a), miR-664b-3p was dramatically down-regulated (P < 0.01) in the colon cancer tissue, suggesting miR-664b-3p plays a significant role in colon cancer progression. Encouraged by this, the normal colon cell line NCM460 and common CC cell lines, including HCT116, SW480, DLD-1, SW620, and SW116, were selected to detect the expression level of miR-664b-3p using qRT-PCR. The result showed that compared with the NCM460, the miR-664b-3p expression level in these CC cells was significantly decreased. Furthermore, the reduction (P < 0.001) of the expression in HCT116 and DLD-1 was more evident than that in other CC cell lines (Figure 1(b)). Thus, HCT116 and DLD-1 were selected for a subsequent experiment.

### 3.2 The proliferation, migration, and invasion of colon cancer (CC) cells were suppressed by miR-664b-3p over-expression

**Figure 2.**
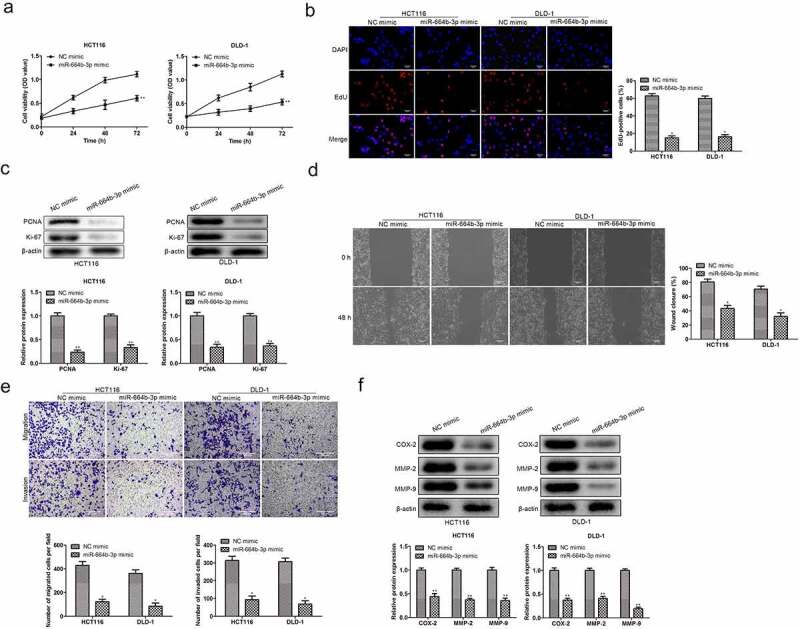
Over-expression of miR-664b-3p regulated the proliferation, migration, and invasion activities of HCT116 and DLD-1. (a) Cell viability was measured by CCK-8, and proliferation was detected by (b) Edu assay. (c) PCNA and Ki-67 expression were inhibited in HCT116 and DLD-1 cells transfected by miR-664b-3p mimics, (d) Wound-healing assay and (e) transwell assay was used to determine the effect of miR-664b-3p on migration and invasion in vitro. (f) The expression level of COX-2, MMP-2, and MMP-9 in overexpressed HCT116 and DLD-1 cells. *P < 0.05, **P < 0.01 vs. NC mimic groups.

To confirm the influence of the expression status of miR-664b-3p on CC cell’s physiological activity, including proliferation, migration, and invasion, we firstly constructed the miR-664b-3p overexpression cell model through transfection miR-664b-3p mimics into HCT116 and DLD-1 cell lines. Then, the cell viability was detected using CCK-8. As shown in Figure 2(a), compared with the cell viability in NC mimics group, CC cells treated with miR-664b-3p mimics significantly suppressed (P < 0.01) the cell proliferation within the measured period, indicating miR-664b-3p over-expression could inhibit the proliferation of CC cells. Meanwhile, the Edu was also used to determine the proliferation in the experiment. As shown in Figure 2(b), the fluorescence intensity in HCT116 and DLD-1 cells treated with miR-664b-3p mimics was significantly reduced (P < 0.05) compared to the fluorescence intensity in NC mimics group, which was in great agreement with the result investigated by CCK-8. Moreover, the Ki-67 and PCNA, the typical proliferation-associated proteins, were determined by Western blot. The result showed that the expression level of these two mitosis-related proteins in cells transfected by miR-664b-3p mimics was markedly decreased (P < 0.01), which is statistically significant (Figure 2(c)).

The relation between CC cells migration and invasion and the expression of miR-664b-3p was determined by wound-healing migration assay and transwell assay. As shown in Figure 2(d), the broader wound width (P < 0.05) was observed in miR-664b-3p overexpressed HCT116 and DLD-1 cells. In line with the data detected by the wound-healing assay, the results from the transwell assay suggested that the high expression level of miR-644b-3p significantly suppressed (P < 0.05) the invasiveness of HCT-116 and DLD-1 cells (Figure 2(e)). Next, the experiment examined the expression level of proteins that impact the migration and invasion of CC cells, and COX-2, MMP-2, and MMP-9 were selected for this purpose. As shown in Figure 2(f), the expression level of these three migration-related proteins was significantly inhibited (P < 0.05) in HCT116 and DLD-1 cells after transfection of miR-664b-3p mimics. The above results illustrated that CC cells’ physiological activity was suppressed by miR-664b-3p over-expression.

### 3.3 miR-664b-3p promoted the apoptosis of HCT116 and DLD-1 cells

**Figure 3.**
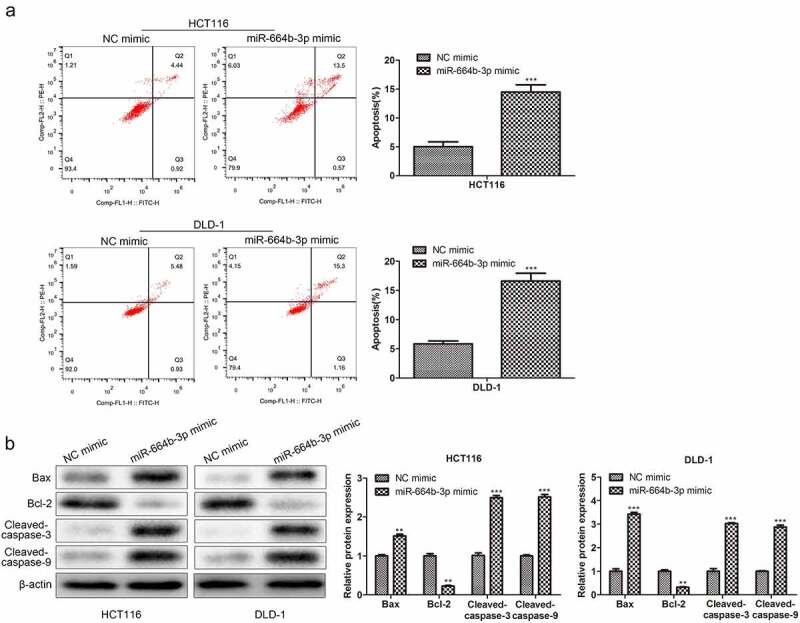
The effect of over-expression of miR-644b-3p on the apoptosis of HCT116 and DLD-1. (a) The proportion of apoptosis cells detected by flow cytometry. (b) Bax, Bcl-2, cleaved-caspase-3, and cleaved-caspase-9 proteins level was assessed by Western blot and normalized to the expression level of β-actin. **P < 0.01, ***P < 0.001 vs. NC mimic groups.

Having confirmed the suppression effect of miR-664b-3p on proliferation, migration, and invasion of HCT116 and DLD-1 cells, the study further assessed the effect of miR-664b-3p on the induction of apoptosis of CC cells in vitro. As shown in Figure 3(a), compared with NC mimics (HCT116: 5.36%; DLD-1: 6.41%), the proportion of apoptosis CC cells transfected by miR-664b-3p mimics (HCT116: 14.07%; DLD-1: 16.46%) was significantly increased (P < 0.001), suggesting overexpression of miR-664b-3p could promote the apoptosis of these two typical CC cells. Based on these results, the study further explored which apoptosis-associated proteins were affected by overexpression of miR-664b-3p, and the Bax, Bcl-2, cleaved-caspase-3, and cleaved-caspase-9 were selected for this object. As shown in Figure 3(b), the expression of Bax, cleaved-caspase-3, and cleaved-caspase-9 was dramatically facilitated (P < 0.01), while the production of Bcl-2 was markedly decreased (P < 0.01) in CC cells transfected by miR-664b-3p mimics. These results collectively indicated that over-expression of miR-664b-3p could promote the apoptosis of HCT116 and DLD-1 through regulating the expression of apoptosis-associated proteins, which the pro-apoptosis proteins were promoted the anti-apoptosis protein was inhibited.

### 3.4 miR-644b-3p directly targeted Budding uninhibited by benzimidazole 3 (BUB3)

**Figure 4.**
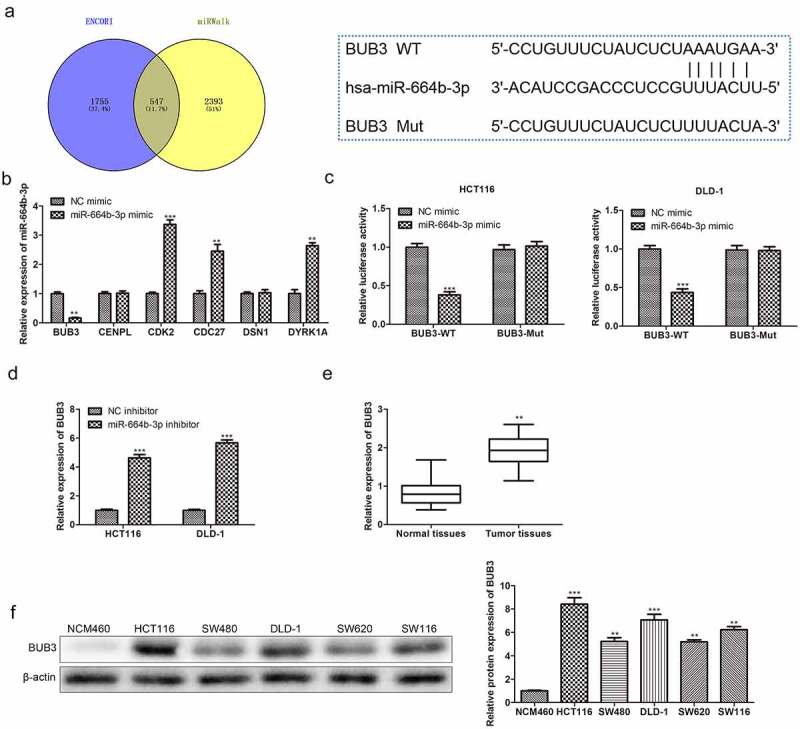
miR-664b-3p specifically regulates the expression of BUB3 protein. (a) ENCORI and miRWalk predicted the target of the miR-664b-3p. (b) The relative expression level of mRNA of different mitogen-related proteins in cells transfected with miR-664b-3p mimics and NC mimics. **P < 0.01, ***P < 0.001 vs. NC mimic groups. (c)Specific regulation effect of miR-664b-3p on the expression of BUB3 was detected using luciferase assay. ***P < 0.001 vs. NC mimic groups. (d) Expression relation between miR-664b-3p and BUB3. ***P < 0.001 vs. NC inhibitor groups. (e) A high expression level was detected in COAD using TCGA. **P < 0.01 vs. Normal tissues. (f) The expression situation of BUB3 protein in CC cell lines, including HCT116, SW480, DLD-1, SW620, and SW116. **P < 0.01, ***P < 0.001 vs. NCM460 cell lines.

To further explore the regulatory mechanism of miR-644b-3p on CC cells, the ENCORI and miRWalk were used to predict the target of miR-644b-3p. The intersection of these two databases was used to pick out cell cycle-related proteins (Figure 4(a)). As shown in Figure 4(b), sex proteins were selected to validate the relation of expression levels between these proteins and miR-644b-3p. Transfection of miR-664b-3p mimic into CC cells only exerted a significant suppression (P < 0.01) effect on the expression of BUB3. Thus, BUB3 protein was used to validate the targeted regulation of miR-644b-3p further using luciferase assay. As shown in Figure 4(c), the result showed that the fluorescence intensity in BUB3 wild type (BUB3 WT) cells was significantly reduced (P < 0.001) when treated with miR-664b-3p mimics. However, there were no fluorescence intensity changes in BUB3 mutated type (BUB3 Mut) cells when transfected with miR-664b-3p mimics, indicating miR-664b-3p could specifically regulate the expression of BUB3.

Furthermore, the regulation effect of miR-664b-3p on the expression of BUB3 was subsequently assessed. HCT116 and DLD-1 transfected with miR-664b-3p inhibitor significantly increased (P < 0.001) the expression of BUB3 (Figure 4(d)), suggesting that low-expression of miR-664b-3p could increase the expression of BUB3. Furthermore, the TCGA database was used in this study to examine the expression level of BUB3 in colon adenocarcinoma (COAD). As shown in Figure 4(e), the expression of BUB3 in COAD was significantly increased (P < 0.01). Furthermore, the different expression levels of BUB3 in COAD based on individual cancer stages were assessed using the TCGA database. The expression level of BUB3 continuously maintained a high expression status from stage 1 to stage 4 of cancer stages, and the highest expression level was detected in stage 1. These results indicated that the expression of BUB3 maintained a high level in COAD. Based on this investigation, the study further explored the expression level of BUB3 in different CC cell lines, including HCT116, SW480, DLD-1, SW620, and SW116. The Western blot result showed that a higher (P < 0.01) expression of BUB3 protein was detected in these five colon cancer cell lines. Furthermore, its expression in HCT116 and DLD-1 cells was higher than in other CC cells (Figure 4(f)). Taken together, miR-664b-3p could negatively regulate the expression of BUB3, which is closely related to the occurrence and development of colon cancer.

### 3.5 Budding uninhibited by benzimidazole 3 (BUB3) reversed the effect of miR-644b-3p on colon cancer cells

**Figure 5.**
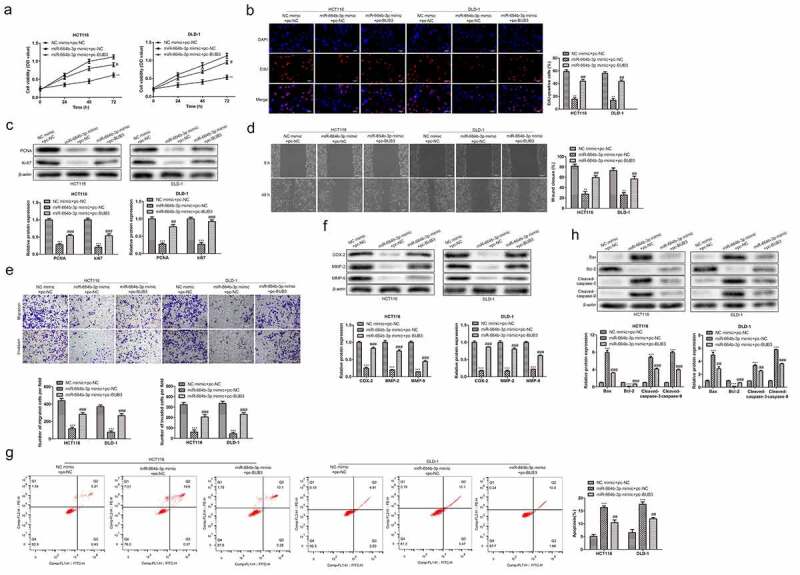
BUB3 protein regulated the tumor-related physiological activity of HCT116 and DLD-1 transfected by miR-664b-3p. (a) BUB3 protein increased the cell viability of miR-644b-3p over-expressed HCT116 and DLD-1. (b). BUB3 protein promotes the proliferation of miR-644b-3p over-expressed CC cells assessed using Edu assay. (c) BUB3 protein boosts the expression of PCAN and Ki67 in miR-644b-3p over-expressed CC cells. (d) Wound-healing assay and (e) transwell assay were used to verify the effect of BUB3 protein on migration and invasion of miR-644b-3p over-expressed HCT116 and DLD-1 in vitro. (f) The expression level of COX-2, MMP-2, and MMP-9 in HCT166 and DLD-1 after transfected by miR-644b-3p mimics and pc-BUB3. (g) Flow cytometry reflected the influence of BUB3 protein on the apoptosis of miR-644b-3p over-expressed HCT166 and DLD-1. (h) Western blot images revealed the effect of BUB3 protein on the expression level of Bax, Bcl-2, Cleaved-caspase-3, and Cleaved-caspase-9 in miR-644b-3p over-expressed HCT116 and DLD-1. **P < 0.01, ***P < 0.001 vs. NC mimic + pc-NC group; ^##^*P* < 0.01, ^###^*P* < 0.001 vs. miR-664b-3p mimic + pc-NC group.

To confirm the effect of BUB3 protein on the physiological activity of HCT116 and DLD-1. The pcDNA-BUB3 and miR-664b-3p mimics were used to transfect HCT116 and DLD-1 together. As shown in Figure 5(a), compared with NC mimics + pc-NC group, cell viability of HCT116 and DLD-1 was significantly decreased when treated with miR-664b-3p mimics and pcDNA-NC (P < 0.01). However, the cell viability after transfecting pcDNA-BUB3 and miR-664b-3p favored an increase (P < 0.05) over reduction compared with CC cells in miR-664b-3p mimics and pcDNA-NC group, suggesting over-expression of BUB3 protein could reverse the inhibition of the cell viability caused by transfecting of miR-664b-3p mimics. Meanwhile, the result from the Edu assay exhibited the same trend as CCK-8, in which the proliferation of HCT116 and DLD-1 was markedly inhibited (P < 0.05) after transfecting with miR-664b-3p mimics and pcDNA-NC. In contrast, the proliferation was significantly boosted when transfected with the pc-BUB3. Noticeably, the fluorescence intensity was significantly increased (P < 0.05) in the pc-BUB3 treated group, which indicated that BUB3 could reverse the inhibition effect of miR-664b-3p on HCT116 and DLD-1 (Figure 5(b)).

Furthermore, proliferation-related proteins, including PCNA and Ki67 was detected again using Western blot. As shown in Figure 5(c), the expression of PCNA and Ki67 was significantly suppressed (P < 0.001) when cells were treated with miR-664b-3p mimics and were dramatically restored (P < 0.001) after transfected with pc-BUB3. These results indicated that BUB3 could promote the proliferation of the HCT116 and DLD-1 overexpression of miR-664b-3p. Next, the wound-healing assay and transwell assay was used to assess the effect of BUB3 protein on the migration and invasion of HCT116 and DLD-1. As shown in Figure 5(d,e), compared with cells transfected by miR-664b-3p mimics and pc-NC, the broadness was markedly reduced (P < 0.01) after cell transfected with pc-BUB3. Furthermore, the Transwell assay exerted the same trend that pc-BUB3 restored (P < 0.001) the invasion activity of the HCT116 and DLD. Likewise, images captured by Western blot showed that pc-BUB3 increased (P < 0.001) the expression of COX-2, MMP2, and MMP-9 in cells that were over-expressed of miR-664b-3p (Figure 5(f)), suggesting that pc-BUB3 could reverse the suppression effect on CC cells migration and invasion caused by overexpression of miR-664b-3p.

Moreover, the study further examined the effect of BUB3 protein on the apoptosis of HCT116 and DLD-1. Flow cytometry showed that the proportion of apoptosis cells in HCT116 and DLD-1 were significantly reduced (P < 0.01) after being transfected by pc-BUB3 (HCT116: 10.38%; DLD-1: 12.06%) compared to the cell transfected by miR-664b-3p mimics and pc-NC (HCT116: 16.97%; DLD-1: 18.57%) (Figure 5(g)). Thus, to further investigate the effect that transfection of pc-BUB3 could inhibit the apoptosis of CC cells induced by overexpression of miR-664b-3p, the experiment measured the expression of proteins closely related to the apoptosis, and the Bax, Bcl-2, Cleaved-caspase-3, and Cleaved-caspase-9 were selected for the purpose. As shown in Figure 5(h), pro-apoptosis proteins were significantly suppressed (P < 0.001) compared with cells transfected by miR-664b-3p mimics and pc-NC. In contrast, the Bcl-2, an anti-apoptosis protein, was markedly increased (P < 0.001) in CC cells transfected by pc-BUB3. The results described above indicated that BUB3 protein could resist the effect caused by overexpression of miR-664b-3p on HCT116 and DLD-1.

## 4. Discussion

Recently, much evidence has been illustrated that miRNA plays a significant role in the development and progression of tumors. Disorders of the expression of miRNAs could cause the dysfunction of the normal cell by influencing the expression of related proteins and then inducing the tumorigenesis [25,26]. Thus, it is a novel and potent strategy for cancer treatment to find a crucial miRNA that is an abnormal expression in the tumor tissue to be used as a biomarker that could screen and even treat cancer, reducing the mortality of the disease [27].

There are little researches about miR-664b-3p in colon cancer. However, the data from the ENCORI database showed that the expression of miR-664b-3p was significantly decreased in the COAD tissue, which was in excellent agreement with the detected expression level in the CC cell lines. The physiological activity of the tumor, including proliferation, migration, and invasion, decides the progression and development of cancer, affecting the prognosis of the disease [28,29]. Thus, the study investigated the influence of over-expression miR-664b-3p on these three features. The result revealed that the proliferation, migration, and invasion of CC cells overexpressed with miR-664-3p was significantly inhibited.

Moreover, the promotion of miR-664b-3p on the apoptosis of CC cells was also observed in the study. Bcl-2 is an essential gene that initiates apoptosis, and Bcl-2 has the functions of inhibiting mitochondrial rupture, regulating intracellular calcium concentration, and suppressing the cell apoptosis via inhibiting the cytotoxicity of Bax [30]. In contrast to the Bcl-2, Bax could induce changes in mitochondrial permeability and activate apoptosis-related proteins, thus promoting apoptosis [31]. Caspase-3 and Caspase-9 are the main effector proteins in cell apoptosis. They are blessed with the ability that destroys extracellular matrix proteins and skeletal proteins while cleaving DNA repair-related molecules [32]. The study revealed that over-expression of miR-664b-3p in CC cells could promote the expression of caspase-3, caspase-9, and Bax, while inhibiting the expression of Bcl-2, suggesting that miR-664b-3p has an irreplicable impact on regulating the balance between proliferation and apoptosis.

ENCORI and miRWalk database, the comprehensive miRNA target gene database which could be used to predict the target gene and protein of miRNA [30,31], was used to explore the mechanism of miR-664b-3p on suppression of CC cells’ physiological activity, and the BUB3 was selected. BUB3, an essential component of the spindle assembly checkpoint (SAC), ensures that all sister chromatids are attached to kinetochore fibers originating from opposite poles of the bipolar spindle before anaphase onset [32,33]. In addition, BUB3 protein plays a vital role in mitosis and participates in embryo development [34]. Overall, it is critical for cell proliferation and differentiation. The luciferase assay results showed that BUB3 was the target protein of the miR-664b-3p, and the miR-664b-3p could inhibit the expression of BUB3.

Furthermore, analysis of the expression level of BUB3 in COAD patients by TCGA validated that the BUB3 was a high expression in the tissue of COAD patients from stage 1 to stage 4. What is more, the expression level of BUB3 was also higher in CC cell lines than that in NCM460. These results suggested that BUB3 deserves further consideration as a biomarker to screen and diagnose colon cancer.

Encouraged by the difference in the expression level of BUB3 protein between normal cells and colon cancer cells, we further investigated the function of BUB3 in the development of the cancer cells. The results showed that BUB3 could alleviate the effect of miR-664b-3p on cells’ viability, proliferation, migration, and invasion. Meanwhile, the BUB3 protein could also reverse the promotion effect of miR-664b-3p on the apoptosis of the HCT116 and DLD-1 while boosting the expression of Bcl-2 and inhibiting the production of Bax, caspase-3, and caspase-9 in colon cancer cells over-expressed of miR-664b-3p. In addition, these results indicated that the increase of the BUB3 expression could attenuate the regulation effect of miR-664b-3p on HCT116 and DLD-1.

## 5. Conclusions

In conclusion, this study demonstrated that miR-664b-3p could serve as a tumor-inhibitor miRNA in the progression of CC via suppressing the expression of the BUB3, which was an abnormal expression in the CC patients’ tissue. In summary, miR-664b-3p/BUB3 axis could be used as a potential therapeutic target in CC.

## Data Availability

The datasets used and/or analyzed during the current study are available from the corresponding author on reasonable request.
